# Prioritization of Unknown LC-HRMS Features Based on Predicted Toxicity Categories

**DOI:** 10.1021/acs.est.4c13026

**Published:** 2025-04-21

**Authors:** Viktoriia Turkina, Jelle T. Gringhuis, Sanne Boot, Annemieke Petrignani, Garry Corthals, Antonia Praetorius, Jake W. O’Brien, Saer Samanipour

**Affiliations:** † Van ‘t Hoff Institute for Molecular Sciences (HIMS), University of Amsterdam, Amsterdam 1090 GD, Netherlands; ‡ Institute for Biodiversity and Ecosystem Dynamics (IBED), University of Amsterdam, 1090 GE, Amsterdam, Netherlands; § Queensland Alliance for Environmental Health Sciences (QAEHS), The University of Queensland, 20 Cornwall Street, Woolloongabba, Queensland 4102, Australia; ∥ UvA Data Science Center, University of Amsterdam, Amsterdam 1000 GG, Netherlands

**Keywords:** nontarget analysis, high-resolution mass spectrometry, prioritization, ecotoxicity, machine learning

## Abstract

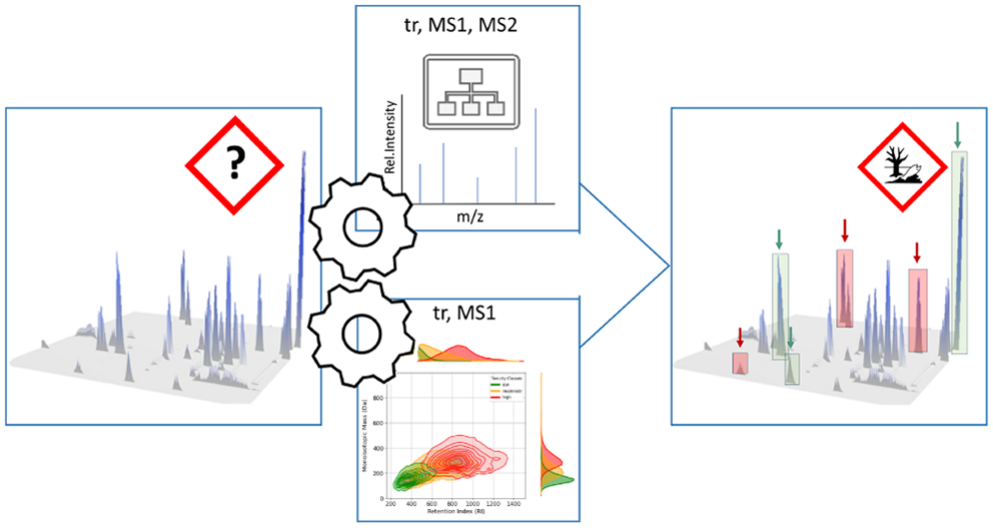

Complex environmental samples contain a diverse array of known and unknown constituents. While liquid chromatography coupled with high-resolution mass spectrometry (LC-HRMS) nontargeted analysis (NTA) has emerged as an essential tool for the comprehensive study of such samples, the identification of individual constituents remains a significant challenge, primarily due to the vast number of detected features in each sample. To address this, prioritization strategies are frequently employed to narrow the focus to the most relevant features for further analysis. In this study, we developed a novel prioritization strategy that directly links fragmentation and chromatographic data to aquatic toxicity categories, bypassing the need for identification of individual compounds. Given that features are not always well-characterized through fragmentation, we created two models: (1) a Random Forest Classification (RFC) model, which classifies fish toxicity categories based on MS1, retention, and fragmentation data—expressed as cumulative neutral losses (CNLs)—when fragmentation information is available, and (2) a Kernel Density Estimation (KDE) model that relies solely on retention time and MS1 data when fragmentation is absent. Both models demonstrated accuracy comparable to that of structure-based prediction methods. We further tested the models on a pesticide mixture in a tea extract measured by LC-HRMS, where the CNL-based RFC model achieved 0.76 accuracy and the KDE model reached 0.61, showcasing their robust performance in real-world applications.

## Abstract 2

This study presents a novel prioritization strategy for LC-HRMS NTA that directly links chromatographic and fragmentation data to the activity of unknown features, bypassing the need for their identification.

## Introduction

The increasing number and variety of produced chemicals hinder the investigation and assessment of their exposure levels and impacts on human and environmental health.^1−3^ Only specific groups of chemicals are routinely monitored as a regulatory measure, thereby limiting the exposure assessment by excluding a diverse mixture of chemicals.^4,5^ In order to investigate chemical exposure realistically, accounting for the diversity of structures and their properties in samples necessitates the application of unbiased approaches.^5−7^

A major portion of contaminants of emerging concern (CECs) is covered by semipolar and polar organic compounds.^8,9^ High-resolution tandem mass spectrometry coupled with reverse-phase liquid chromatography (LC-HRMS) is a well-known technique for analyzing such chemicals.^9,10^ To limit the bias toward a small set of standardized analytes, the LC-HRMS nontargeted (NTA) approach has been successfully developed and employed in environmental studies.^11−16^ NTA does not require prior knowledge of the chemicals present in a sample and enables the analysis of all detectable compounds. However, the complex nature of environmental samples results in highly convoluted data, requiring careful and thorough preprocessing.^17−20^ To support this, both vendor software, such as Compound Discoverer, and open-source software, including patRoon^21^ MS-DIAL^22^ and MZmine,^23^ have been developed for data processing. Typically, the number of detected features in NTA—data points constructed by retention time, chromatographic peak intensity, and *m*/*z* of precursor ions—often counts in hundreds or thousands, though typically fewer than 5% of these can be identified.^19,24−26^

Confident identification of detected features requires considerable computational and research resources.^17,26,27^ Therefore, prioritization is used as a strategy to focus the available resources on the most relevant features for a given study’s objectives.^28^ The prioritized species of interest undergo a more detailed and thorough investigation. There are two ways to prioritize: online and offline. Online prioritization refers to a variety of parameters used to acquire the data, e.g., to perform data-dependent acquisition.^28^ On the contrary, offline prioritization strategies employ post hoc analysis of MS1 and MS2 (both data-dependent acquisition (DDA) and data-independent acquisition (DIA)) data, including intensity-based prioritization, statistical analysis, structural evaluation, and quantitative structure–activity relationship (QSAR) evaluation.^6^ Both approaches can be advantageous, depending on the scope of the study. The online approach provides less convoluted data with higher sensitivity, making it ideal for targeting specific compounds of interest. However, it carries the risk of missing highly relevant but initially unknown features. For example, low-intensity compounds might be detected in MS1 but overlooked during MS2 acquisition. This omission can result in the loss of valuable information necessary for further characterization. In contrast, the offline approach prioritizes features of interest while preserving the full sample information. This is particularly beneficial for environmental studies as the data can be revisited for retrospective analyses, such as identifying previously unrecognized toxic or persistent compounds.^29^

Environmental studies primarily focus on the properties and potential activities of detected chemicals, including their ecological toxicity.^30,31^ As a result, prioritization strategies often aim to highlight features that may pose significant (eco)toxicological risks.^32^ For example, Peets et al. developed the MS2Tox R package, which uses a regression model to predict toxicity (-[LOG(mM)]) for organisms like fish, water fleas, and algae.^33,34^ Similarly, Rahu et al. developed an algorithm to predict endocrine-disrupting activity using binary or multilabel classification,^35^ while Arturi and Hollender created MLinvitroTox, a tool to classify chemicals as toxic or nontoxic based on nearly 400 target-specific and over 100 cytotoxic end points.^36^

All these studies rely on predicting molecular formulas and fingerprints from MS2 spectra using tools like CSI:FingerID/SIRIUS.^37^ Therefore, the accuracy of these models is closely tied to the precision of the predicted molecular fingerprints. Incorrectly calculated fingerprints can significantly distort the predictions, and this accuracy can be compromised by various experimental factors, such as incomplete fragment detection, false-positive fragments, or missing characteristic fragments due to instrumental noise. Bypassing the molecular fingerprint prediction and instead predicting chemical activity directly from chromatographic and MS data potentially reduces this uncertainty and enhances the interpretability of the results.

Aquatic toxicity has been shown to be related to the hydrophobicity of the chemicals, their molecular weights, and structural alerts.^38−40^ Each detected feature indirectly includes structural information: chromatographic retention times and mass spectral (MS) information, such as accurate mass (MS1) and fragmentation spectra (MS2). Therefore, this information can be used to assess the toxicity of the chemicals without any knowledge of their molecular structure, formula, or fingerprint.

In this study, we developed a prioritization strategy based on acute aquatic toxicity using chromatographic and fragmentation information on unknown features. Instead of identifying each feature, we directly described their activity without any knowledge of the molecular structure. We constructed two models: a Random Forest Classification (RFC) model to assign toxicity categories based on fragmentation data and a Kernel Density Estimation (KDE) model to determine the probability of features belonging to specific toxicity classes. These algorithms allow us to characterize unknown features at both the mass spectral and chromatographic levels. The performance of the constructed models was evaluated using both spectra from databases with measured toxicity values and tea extracts spiked with 253 pesticide standards.

## Methods

Since fragmentation information in NTA is often incomplete, we developed a probabilistic algorithm to predict toxicity categories that relies solely on chromatographic data, specifically retention time and MS1 spectra. This algorithm uses KDE in both the retention and mass domains to identify regions of chromatograms that are more likely to correspond to higher or lower toxicity, effectively mapping the toxicity. Additionally, in the presence of reliable fragmentation data, the CNL-based RFC model can be applied to categorize toxicity based on detected fragments.

### Overall Workflow

**Figure 1:**
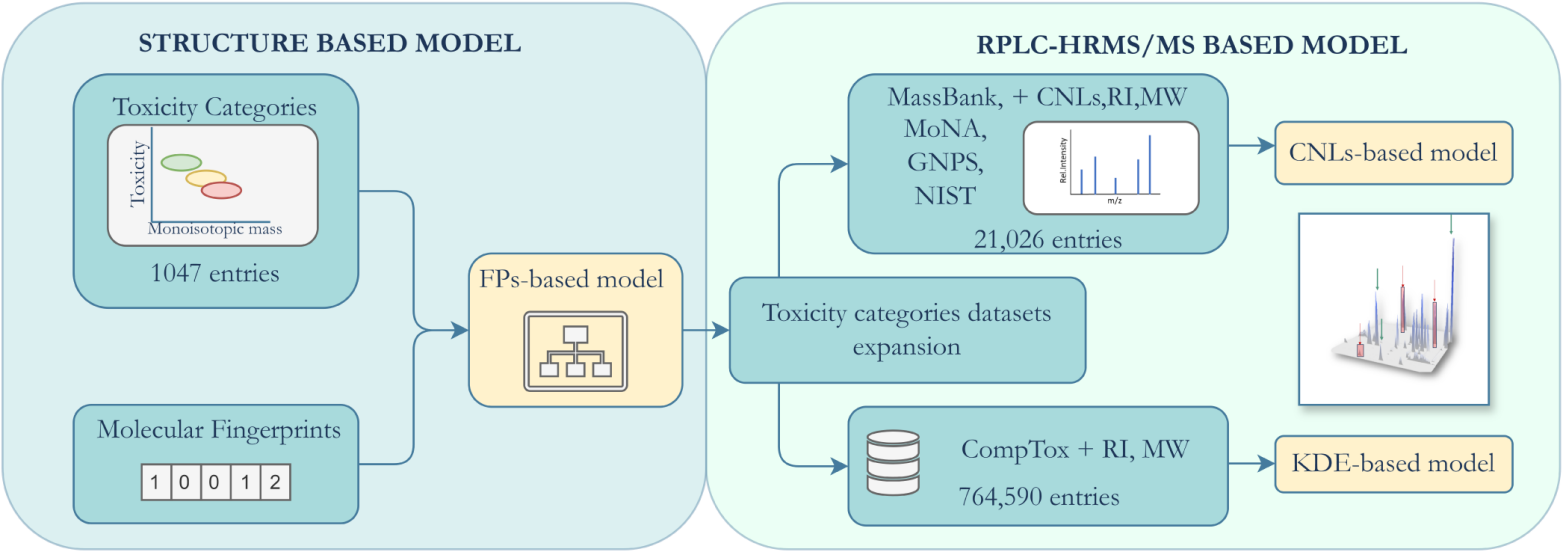
Overall workflow. A structure-based model was developed using a fish toxicity data set and molecular fingerprints (FPs). This FP-based model was then applied to the CompTox and Spectral databases, incorporating additional information such as molecular weight (MW) and retention indices (RI). A KDE-based model was constructed based on this data. Furthermore, fragmentation information (i.e., characteristic neutral losses, CNLs) was integrated to develop a CNL-based model.

To develop the models, we employed a semisupervised approach. First, a structure-based classification model was built using an experimentally generated acute fish toxicity data set and molecular fingerprints. This model was then used to expand toxicity knowledge by predicting the toxicity category for chemicals from large, existing spectral and environmentally relevant databases. The CompTox data set^41^ was used to develop a KDE-based model, while for the CNL-based model, HRMS spectra were collected from sources such as MassBank, MoNA, GNPS, and NIST.^42−45^ Finally, the results of the developed models were compared and validated using an experimentally acquired pesticide mixture in a tea extract (Figure 1).

### Data Sets

For the model development, validation, and testing, we employed four different data sets, namely: a set of experimental acute toxicity values, referred to as the fish toxicity data set, the CompTox data set, a Cumulative Neutral Loss (CNL) data set, and experimentally acquired LC-HRMS chromatogram of a standard pesticide mixture in tea extracts, referred to as the pesticide mixture data set.

#### Fish Toxicity Data Set

The selection of the ecological species and the end point was mainly driven by the size of the curated data available. We gave priority to model accuracy and robustness rather than ecological coverage. Consequently, we focused on experimentally defined LC 50 values for fathead minnows (*Pimephales promelas*), which today is the largest and most curated data set following the OECD protocol. In this study, the data set was compiled from two sources: Cassotti et al.^46^ and Schür et al.^47^ These sources are themselves compilations of numerous empirical studies relying on data collected from a wide range of experiments. The data set includes concentration values, expressed in LOG(mg/L), representing the dose that causes 50% mortality in fathead minnows over a 96-h exposure period (LC 50). In total, 1047 chemicals in the data set come from various chemical families, including pharmaceuticals, pesticides, conventional persistent organic pollutants, and industrial chemicals.^48^ Due to the inherent variability in *in vivo* experiments, where results can vary even under identical conditions, the data set includes multiple repeated measurements for each chemical. The median value from these repetitions was used as the reference LC 50 value for each compound.

#### The CompTox Data Set

The CompTox Chemicals Dashboard, developed by the U.S. Environmental Protection Agency (EPA), is a vast database that has been curated over 15 years through both manual and automated efforts as part of the EPA’s DSSTox project. It contains detailed information on 764,590 chemicals (as of March 2024), including identifiers such as CAS numbers and SMILES, synonyms, and key properties such as solubility, logKow, and molecular weight. Additionally, it provides data on physicochemical characteristics, environmental fate, exposure, usage, and available toxicity results from both *in vivo* and *in vitro* studies. For our purposes, the CompTox data set was filtered to exclude salts, metalloids, and compounds containing elements not typically found in fish toxicity data sets or not analyzable via reverse-phase liquid chromatography (RPLC), leaving only chemicals composed of H, B, C, N, O, F, Si, P, S, Cl, Br, and I.

#### CNL Data Set

The CNLs were used in place of fragments to reduce the dimensionality of the data set while retaining the structural information embedded in the spectra. The CNL data set was generated from entries in the MassBank EU, MoNA, GNPS, and NIST20 databases. These entries were obtained using electrospray ionization (positive mode) with a mass resolution of ≥5000 and proton adducts and included data from various mass analyzers (e.g., Q-TOF and Orbitrap) with different collision energies. To ensure data quality, only spectra with at least three recorded fragments were included. When multiple spectra were available for a single compound, they were merged using a ± 0.01 Da mass window, yielding 21026 unique spectra. Rare fragments, present in only a single instance across multiple entries, were discarded to focus on more representative data.

For each compound, the CNLs were calculated by subtracting the fragment *m*/*z* values from the precursor ion mass. These CNLs were then converted into bit vectors representing masses from 0 to 1000 Da with a step size of 0.01 Da (±5 mDa mass tolerance). In this representation, a value of 1 indicated the presence of a CNL in the spectrum, while a value of 0 indicated its absence. To further refine the data set, CNLs that occurred in fewer than 100 spectra were removed, resulting in a final data set of 2440 common CNLs, with mass values ranging from 1.03 to 382.19 Da.

In addition to the CNL bit vectors, the monoisotopic mass and predicted retention index were added as continuous features. To calculate the monoisotopic mass, the mass of proton, 1.008 Da, was subtracted from the measured *m*/*z* value of the proton adduct. To predict retention indices, the previously reported model by van Herwerden et al.^49^ based on molecular fingerprints and retention indices calculated on the cocamide scale was employed.^50^ The retention indices are less affected by variations in chromatographic conditions because they are relative to the retention time of internal standards.^49−51^ Therefore, this combination of features allowed us to incorporate key structural and chromatographic information into the model while minimizing the number of variables. To interpret the importance of individual CNLs, a search was conducted in the CompTox Chemicals Dashboard to identify relevant substructures. The prominent substructures frequently identified in the search results were used to inform the model interpretation.

#### Pesticide Mixture Data Set

The prioritization models were further tested on experimentally acquired data sets of varying levels of tea extract matrix spiked with 253 pesticide standards. Tea extract was used as a complex matrix instead of drinking water to assess the algorithm’s performance, considering its impact on ionization, fragmentation, and deconvolution. Pesticides, relevant to aquatic toxicity and included in the EU Zero Pollution Action Plan, were spiked into the extract at 10× and 100× dilution factors, and the detected standards were used to test the algorithm.

The samples were analyzed using the previously reported RPLC-ESI-Q-TOF method.^52^ The three standard mixtures of pesticides from Neochema were diluted with a filtered water/ethanol solution (50%:50%, v/v, Blank) or filtered tea extract, which was further diluted 1:10 or 1:100 with Blank to achieve final analyte concentrations of 100 μg/L. Each sample was analyzed twice. A detailed description of the samples, acquisition method, and data processing is provided in Section S1.

To obtain features and fragmentation information on detected pesticides, suspect screening was performed by applying the Universal Library Search Algorithm (ULSA).^53^ A suspect list of the pesticides was compiled from the MS2 spectra found in the MassBank EU, MoNA, and NIST20 databases, with a resolution higher than 5000 for each of these chemicals. To minimize the detection of false-positive fragments, a mass tolerance of 0.010 Da and a minimum MS1 intensity of 2000 were applied for matching acquired fragments with suspects. Additionally, to increase the confidence in correctly detected fragments, a retention time tolerance of 0.5 min was employed for the comparison of MS1 and fragment profiles.

The detected standards were used to test the developed algorithms. For that purpose, first, retention times were converted into retention indices based on the cocamide series. A linear regression model was built using the retention times of 18 detected pesticides across all six chromatograms and their predicted retention indices (Figure S3). The model was then applied to the rest of the detected pesticides (eq S2). Finally, the developed models were applied to the detected suspects to assign toxicity categories. Since the 96-h LC 50 mortality of fathead minnows was unknown for the detected pesticides, the predicted toxicity categories based on the structure presented via molecular fingerprints were used as benchmark toxicity categories.

### Modeling

#### Assigning Toxicity Categories

A previous study demonstrated the advantages of using clustering algorithms as an alternative to expert-based toxicity categorization, which typically relies on fixed LC 50 or EC 50 thresholds.^48^ These expert-defined thresholds often fail to account for the uncertainty in LC 50 measurements or the structural similarities between chemicals. The k-means clustering algorithm, an unsupervised iterative method, clusters data based on the distances between measurements and a set of user-defined centroids. This approach takes advantage of structural similarities between chemicals when forming clusters.

In this study, we applied k-means clustering to categorize chemicals from a fish toxicity data set, using scaled LC 50 values [LOG(mg/L)], monoisotopic mass, and six elemental mass defects (EMDs): CO, CCl, CN, CS, CF, and CH as input features.^54^ Since LC 50 values primarily determine toxicity while structural information captures relationships between chemical structures, we assigned a weight of 1/7 to each of the monoisotopic masses and the six EMDs. This ensured that LC 50 values and structural information contributed equally.

The algorithm converged after 12 iterations with an inertia of 323, forming three distinct clusters (Figure S1). These clusters were then labeled as low, moderate, and high toxicity based on their positions along the *y*-axis (LC 50 values). The final clustering allowed clear differentiation between low toxicity (*n* = 238), high toxicity (*n* = 202), and moderate toxicity (*n* = 467) chemicals, providing a robust categorization informed by both toxicity data and structural characteristics.

#### Classification Models

For modeling, a Random Forest Classifier (RFC) algorithm was implemented in Julia with scikit-learn.^55^ Random Forest is a supervised algorithm that constructs several unique decision trees from bootstrap data. After model development, the major voting of predictions resulting from the individual trees is used to produce the final RFC model prediction.^56^ RFC has been successfully applied in previous QSAR modeling studies, demonstrating its accuracy.^48,52,57,58^ A key advantage of RFC is its ability to model complex, nonlinear relationships while maintaining better interpretability compared to many advanced machine learning models.^56^ This balance of robustness and explainability makes RFC a suitable and reliable choice for this study

#### FP-Based Model

To train the fingerprint-based (FP-based) model, the fish toxicity data set (*n* = 1047) was split into a training set (*n* = 854), test set (*n* = 95), and global test set (*n* = 98) via stratified sampling (Figure S1). The input for the classification model was the optimizied fingerprint^59^ and the output was toxicity categories. The molecular fingerprints were calculated based on six different nonhashed molecular fingerprints, namely, Atom Pair 2D Count (AP2DC),^60^ Electrotopological State (E-state),^61^ Klekotha-Roth Count (KRC),^62^ Molecular Access Systems (MACCS),^63^ PubChem,^64^ and Substructure Keys Count (SSC) FPs.^65^ A total of 340 bits out of the combined 7073 were selected based on relevance to acute fish toxicity prediction.^59^ The model was optimized on a training set, validated with a test set, and finally tested with a global test set. Hyperparameter optimization for the RFC model was performed via grid search, varying the number of estimators between 100 and 400 with a step of 100, minimum samples per leaf between 2 and 10 with a step of 2, maximum number of features as sqrt or log2, and maximum depth between 10 and 20 with a step of 2. Cross-validation of the hyperparameter optimization was 3-fold. This FP-based model then was utilized to predict the toxicity categories for CompTox and CNL data sets.

#### CNL-Based Model

To train the CNL-based model, the CNL data set was randomly split into a training set (*n* = 18835), test set (*n* = 2093), and global test set (*n* = 98). The input for the classification model consisted of the CNL values converted to a bit vector, monoisotopic mass, and predicted retention indices. The output of the CNL-based model was the predicted probability of assignment to toxicity categories. This probability was further converted into the predicted category based on the defined threshold. The threshold was calculated based on the difference in probabilities between all three categories using only the test set. Hyperparameter optimization was performed in the same manner, as described in Fingerprint-based model. Additionally, to account for class imbalance, the weighted version of RFC was employed. The inverse toxicity class frequency was used to assign higher weights to underrepresented classes in the training set (eq S1). This approach mitigates the data imbalance issue with minimum data structure manipulation, contrary to under- or oversampling strategies.

#### KDE-Based Model

The KDE algorithm^66^ was used for toxicity category mapping on chromatographic dimensions using monoisotopic mass, predicted retention indices, and toxicity categories. KDE is a nonparametric method used for estimating the probability density function (PDF) of a random variable. It is known to be able to model complex, multimodal (e.g., heterogeneous) distributions, handle irregular data, and provide interpretable results. This makes it highly advantageous for this study as it effectively handles data with unknown or nonparametric distributions. Given the fitted KDE, we calculated the probability for each chemical to belong to one of the predetermined toxicity categories using the following formula:
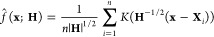
1where *f̂*(**x**) represents the estimated density at point *x*, *n* is the number of data points, *d* is the number of dimensions, *H* is the *d* × *d* bandwidth matrix, *K* is the multivariate kernel function, |**H**| is the determinant of the bandwidth matrix *H*, and **H**^–1/2^ is the matrix square root of the inverse of *H*. Because the classification data are unbalanced, the number of samples in each class was used as weights. This effectively cancels out the 1/*n* in the formula and allows for comparison between the kernel densities.

Bandwidth selection was carried out using Silverman’s rule of thumb:
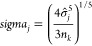
2where σ_*j*_ denotes the standard deviation of the *j*th feature in class *C*_*k*_. This method ensures a smooth estimation of the density function, aiding in the effective prediction of the toxicity classifications.

To evaluate the performance of the KDE-based model, densities for all three toxicity classes were calculated for the fish toxicity data set as only available experimentally measured values. The toxicity category was assigned based on the highest calculated probability (density).

#### Applicability Domain Calculations

The Applicability Domain (AD) defines the parameter space within which a predictive model is expected to make reliable predictions. In this study, we utilized the leverage approach to define and evaluate the AD of our predictive model.^67^ The AD was assessed by calculating the leverage for each sample using the equation:
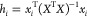
3where *x*_*i*_ is the vector of predictor variables for the *i*th sample, and *X* is the matrix of predictor variables for all training samples. The leverage values indicate the influence of each sample on the model’s predictions.

The warning leverage threshold h* is determined to identify samples that have high leverage and may lead to unreliable predictions. The threshold is typically set to three times the average leverage in the training set;
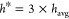
4

Thus, predictions for samples with leverage values greater than these thresholds are considered less reliable and are flagged as outside the AD. To ensure that these do not affect the kernel density estimation, the weights for each sample were set at 1/*h*.

#### Performance Assessment

For the evaluation of the performance of the multiclass classification model, several metrics were computed, including precision, recall, F1 score, and accuracy. The classification metrics provide an overview of the model’s performance for each class. The calculations for these metrics are as follows:

Precision: The ratio of correctly predicted positive observations to the total predicted positives.
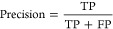
5

Recall (Sensitivity): The ratio of correctly predicted positive observations to all observations in the actual class.
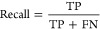
6

F1 Score: The weighted average of Precision and Recall.
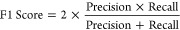
7

Accuracy: The ratio of correctly predicted observations to the total observations
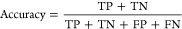
8

To further validate the classification models, the confusion matrix was computed, which provided a detailed breakdown of the model’s predictions compared to the actual class labels. Because of the class imbalance, the confusion matrix was normalized to represent fractions, making it easier to interpret the proportion of correct and incorrect predictions for each class.

### Calculations

All calculations were performed on a personal computer (PC) with an Intel Core i7–1260P central processing unit and 32 GB of RAM operating Windows 10 Education, version 22H2. All of the data processing and statistical analyses were performed using Julia language version 1.6.7. The computational algorithms were computed using ScikitLearn.jl version 0.7 library. The fish toxicity data set, the filtered CompTox data set, and the pesticide mixture data set are publicly available at 10.5281/zenodo.14188167. Additionally, a script to perform the calculations can be accessed at https://bitbucket.org/viktoriiaturkina/toxmap_prioritization.jl/src/main.

## Results and Discussion

In this study, we developed two classification algorithms, machine learning and probabilistic, to predict fish toxicity categories based only on chromatographic and fragmentation data without identified structural information. Additionally, to assign the toxicity categories, we trained the classification model based on molecular FPs. For the probabilistic algorithm, the CompTox database was mapped onto RPLC-HRMS space, and a KDE was fitted to each toxicity category assigned based on the known chemical structures (FPs). For the machine learning algorithm, the RFC model was trained on precursor ion mass (proton adduct), retention indices, and fragmentation information in the form of a CNL bit vector.

### FP-Based Model

The fingerprint-based model developed in this study was an RFC model trained on 900 structures described with optimized molecular fingerprints^59^ and their experimental LC 50 [LOG(mg/L)] values.^46−48^ Optimized hyperparameters of the model were determined as 300 estimators, a minimum of 10 samples per split, a maximum depth of 20, and sqrt as the number of features for the split. To assess the performance of the model, a confusion matrix, accuracy, precision, recall, and F1-score for each toxicity category were calculated (Table 1).

**Table 1:** Performance Assessment of the Fingerprint-Based Classification Model

	Class	Precision	Recall	F1-Score	Support	Accuracy
Training set	Low	0.90	0.83	0.87	263	0.86
	Moderate	0.83	0.94	0.88	446	
	High	0.92	0.66	0.77	145	
Test set	Low	0.72	0.75	0.73	24	0.75
	Moderate	0.81	0.79	0.80	58	
	High	0.54	0.54	0.54	13	
Combined data set	Low	0.88	0.83	0.85	287	0.85
	Moderate	0.83	0.93	0.87	504	
	High	0.88	0.65	0.75	158	

The accuracy of the optimized model was 0.86 for the training set, 0.75 for the test set, and 0.70 as the mean accuracy of 3-fold cross-validation, which was comparable to previously reported QSAR models for toxicity predictions.^68,69^ Performance assessment of the prediction results for chemicals from the test set showed that the model consistently performed well for low and moderate toxicity categories, with F1-Scores of 0.80 and 0.73, respectively . However, the F1-Score for the high toxicity category was lower compared with the other two categories, at 0.54. Based on the confusion matrix and leverage analysis (Figure S4), the lower recall for the high toxicity class in the test set was primarily due to the misclassification of compounds with relatively low leverage values into the moderate toxicity class. Compounds with lower leverage generally have lower monoisotopic mass and show greater similarity to compounds in the moderate and low toxicity categories, which influences the accuracy of high toxicity category predictions. Moreover, the chemicals assigned to the high toxicity category exhibited greater variability in structural representation, with the smallest number of chemicals included: 145 for the training set and 13 for the test set.

To ensure a broader applicability domain for predictive purposes, the final fingerprint-based classification model was trained by using a combination of the training and test sets. A separate global test set containing measured toxicity values, and available LC-HRMS data were reserved for the final model assessment. The validated model was then used to predict toxicity categories for the CompTox and CNL data sets, expanding the data set and supporting the development of a probabilistic approach and the training of a CNL-based model.

### KDE-Based Model

To create a KDE algorithm, the toxicity categories and retention indices were predicted for each chemical from the CompTox data set based on their structures. This information was then used to map toxicity regions within the RPLC-MS domain, linking toxicity categories to features in the chromatogram.

For quality assurance of the KDE-based model, we first filtered the CompTox data set, as described in the Methods section and retained only chemicals that are potentially analyzable by RPLC. Then, to account for the applicability domain of the retention index prediction model, we applied weights based on the calculated leverage of the compounds. This combination minimizes the impact of individual structures that may not have been well represented by the training sets of our models.

**Figure 2:**
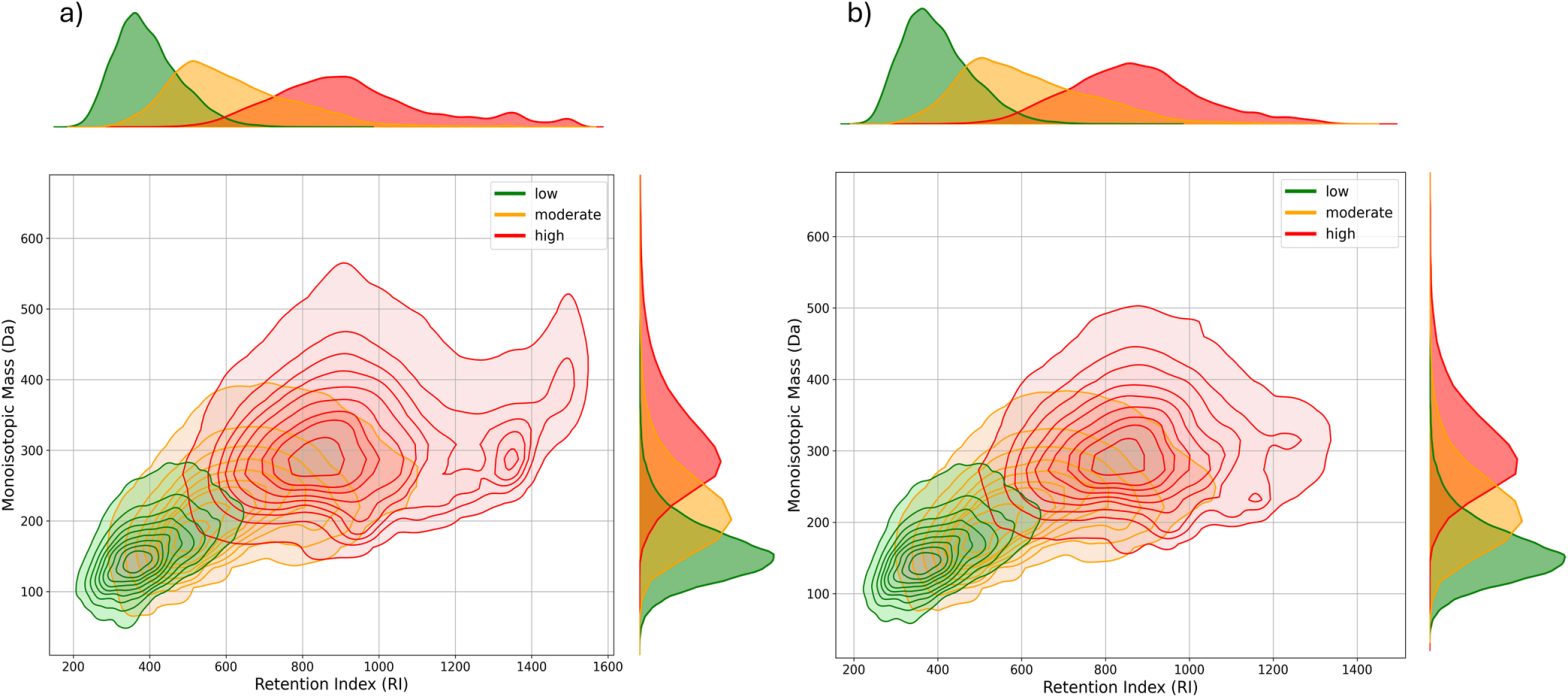
(a) KDE joint plot of the CompTox data set, including only chemicals analyzable by RPLC. (b) KDE joint plot of the CompTox data set, adjusted using calculated leverage weights.

Out of the 764,590 chemicals in the filtered Comptox database, 83,969 chemicals (10.9%) were classified as low toxicity, 458,302 chemicals (59.9%) as moderate toxicity, and 222,319 chemicals (29.0%) as high toxicity. However, only 20.8% of the CompTox data set is well represented by the training set, meaning the calculated leverage for these chemicals is less than the leverage threshold, which is three times the mean value of leverage in the training set, 1.02. To decrease the uncertainty of KDE, we used calculated leverage values as weights to account for the less certain predictions of the fingerprint-based model. Therefore, chemicals that were well represented in the training set gave more weights for the density estimation and more accurately represented the results. The weights used were 1/*h* with *h* being the leverage of a compound (Figure 2a).

The CompTox data set contains a wide variety of structures, with the range of monoisotopic mass between 12.0 and 5,628.0 Da and the predicted retention indices from 178.6 to 1,530.5 RI values (Figure S2). A portion of this space cannot be analyzed with RPLC, either due to insufficient retention (e.g., highly hydrophilic chemicals, often with low masses, i.e., < 70 Da) or excessive retention (e.g., highly hydrophobic chemicals with high mass and high retention indices). Several recent studies have investigated algorithms for assigning chemicals to be analyzable with RPLC or other types of chromatography.^49,70−72^ We applied the machine learning algorithm developed by van Herwerden et al. to the CompTox data set to filter out chemicals that will not be analyzed and therefore detected using RPLC. Of the 671,395 chemicals predicted to fall within the RPLC space, 78,087 (11.6%) were classified as low toxicity, 431,089 (64.2%) as moderate toxicity, and 162,219 (24.2%) as high toxicity. Thus, mainly compounds predicted as highly toxic fell outside the RPLC space (Figure 2b).

The overall accuracy of the algorithm was 0.69. Similar results were observed for overall precision, recall, and F1-score. The metrics of predictions for moderate and high toxicity categories showed comparable performance with precision, 0.75–0.76, recall, 0.6–0.64, and F1-score, 0.67–0.70. Results for low toxicity group with 0.58 for precision and 0.84 for recall differed from the results for the rest of the categories. Overall, the algorithm showed consistently good performance among all categories.

Only 1.3% of chemicals from the low toxicity group were classified as highly toxic chemicals, and 9.4% of high toxicity chemicals were classified as low toxicity chemicals. This indicates that low toxicity compounds generally do not occupy the region of high toxicity class compounds. The larger error occurs in misclassification between moderate and low toxicity groups (28%) and between high and moderate toxicity groups (30%) (Figure S5). The developed algorithm based on KDE was as accurate as the FP-based model, even though no actual structural information was provided to it.

### CNL-Based Model

For CNL-based model construction, we predicted toxicity categories and retention indices for the CNL data set. Out of 20,928 chemicals in the data set, 3,152 chemicals were predicted as low toxicity chemicals, 12,662 as moderate toxicity, and 5,114 as high toxicity. The number of chemicals assigned to different groups shows an imbalance in the data set; therefore, to train the model, the weighted version of RFC was employed.

The optimized CNL-based model showed an overall accuracy of 0.76 for the training set and 0.72 for the test set. Therefore, the final model showed a high level of accuracy comparable to the fingerprint-based classification model. Moreover, precision, recall, and F1-score for the training and test sets showed consistent results for all three toxicity groups (Table S2), indicating excellent performance of the model regardless of the class imbalance.

This model consisted of 600 trees, a minimum of two samples in each leaf, a maximum depth of 20, and ″sqrt″ as a maximum number of features. A difference in probability threshold of 0.08 was applied to the classification results: if the difference in predicted probabilities across all three toxicity categories was less than 0.08 (or 8%), then the chemical was assigned to the moderate toxicity category. This is because, in the KDE model, the moderate toxicity category is the most uncertain and tends to overlap with both the low and high toxicity categories.

The model explained 85% of the variance using 592 variables, including CNLs with masses ranging from 2.01 to 354.19 Da. Based on the impurity decrease across trees, the largest contributions to the model came from the retention index (RI) and accurate mass, cumulatively accounting for 36%. These were followed by contributions from CNLs with masses such as 17.03 Da (1.7%), likely corresponding to NH_3_, 45.01 Da (CH_3_NO), 27.01 Da (HCN), and 63.03 Da (HNO_2_ or CH_2_SO), each contributing around 1%. While these structures are plausible, alternative small molecules corresponding to these masses cannot be disregarded. Higher mass CNLs had an additional contribution, including 294.11 Da, potentially representing multiple aromatic rings or benzyloxy substructures, 216.13 Da, possibly linked to aromatic heterocycles like indole or quinoline, and 174.11 Da, which may correspond to a thiocarboxylic ester. This indicates that the model primarily relies on MS1 data, supplemented by structural information that links to the potentially toxicity-relevant substructures.

### Comparison of the KDE- and CNL-Based Models

Based on the confusion matrices for the training and test sets, a comparable performance to KDE algorithm was observed. The main misclassifications occurred in the moderate toxicity group, while low toxicity was barely ever classified as high toxicity group (1%) and vice versa (0%) (Figure S6). However, the test and training sets for the KDE model and CNL-based model were different; therefore, one-to-one comparison cannot be performed using these data sets. Thus, to further evaluate and compare the prediction power between all three generated models, we used a global test set. These 98 entries had both the CNL bit vector and fish toxicity experimentally determined values and were completely unknown to any of the models.

When the KDE and CNL-based models are compared with the FP-model, the confusion matrices indicate that the FP-based model produced comparable results in predicting low and high toxicity categories. This is a significant outcome, given that the KDE- and CNL-based models do not account for the structural representation of the compounds and are directly linked to chromatographic and mass spectrometry data. However, FP-based model outperformed the other models in predicting the moderate toxicity group, achieving an accuracy of 0.88 compared to 0.47 for the KDE model and 0.43 for the CNL model (Figure S6). This can be explained by the substantial overlap in the RPLC-MS space assigned to the moderate toxicity category, which has a mass range of 110.11 to 336.30 and retention indices between 285.28 and 1139.23, with the regions for low and high toxicity categories.

To further demonstrate the applicability of the developed approach, we applied it to an experimentally acquired data set of a standard pesticide mixture at varying concentrations in a tea extract. Between 141 and 155 pesticides were detected out of 253 in the standard mixture across all three levels of the tea matrix (no tea, 100-fold dilution, and 10-fold dilution). The minimum number of 141 pesticides was detected in the 100-fold diluted tea, while 155 were detected in the 10-fold diluted tea. No clear trend was observed between the number of detected pesticides and matrix dilution. Additionally, the number of detected and matched fragments was compared across the three matrices. The median number of fragments per pesticide was 12 to 13, with the 25th and 75th percentiles ranging from 7 to 21–23. Overall, no significant influence of the matrix level was observed on the number of detected pesticides or the number of corresponding fragments. Since matrix effects and other parameters can influence not only the number but also the quality of detected fragments, the CNL-based model was applied independently to each chromatogram (Table S3 and Figure S7).

To keep prediction results comparable between the models based on KDE and CNLs, we retained only the features corresponding to the standard pesticide mixtures detected in all samples. As part of the ULSA workflow, Suspect screening was applied to identify corresponding features at both the chromatographic and fragmentation levels. In total, 84 pesticides were detected in all three different matrices, 18 of which were used to establish a correlation between retention time and retention indices, yielding an *R*^2^ = 0.91. The remaining 66 pesticides were used for further testing. Based on molecular structure, the FP-based model classified the 66 pesticides as follows: 3 were assigned to the low toxicity category, 48 to moderate toxicity, and 15 to high toxicity.

**Figure 3:**
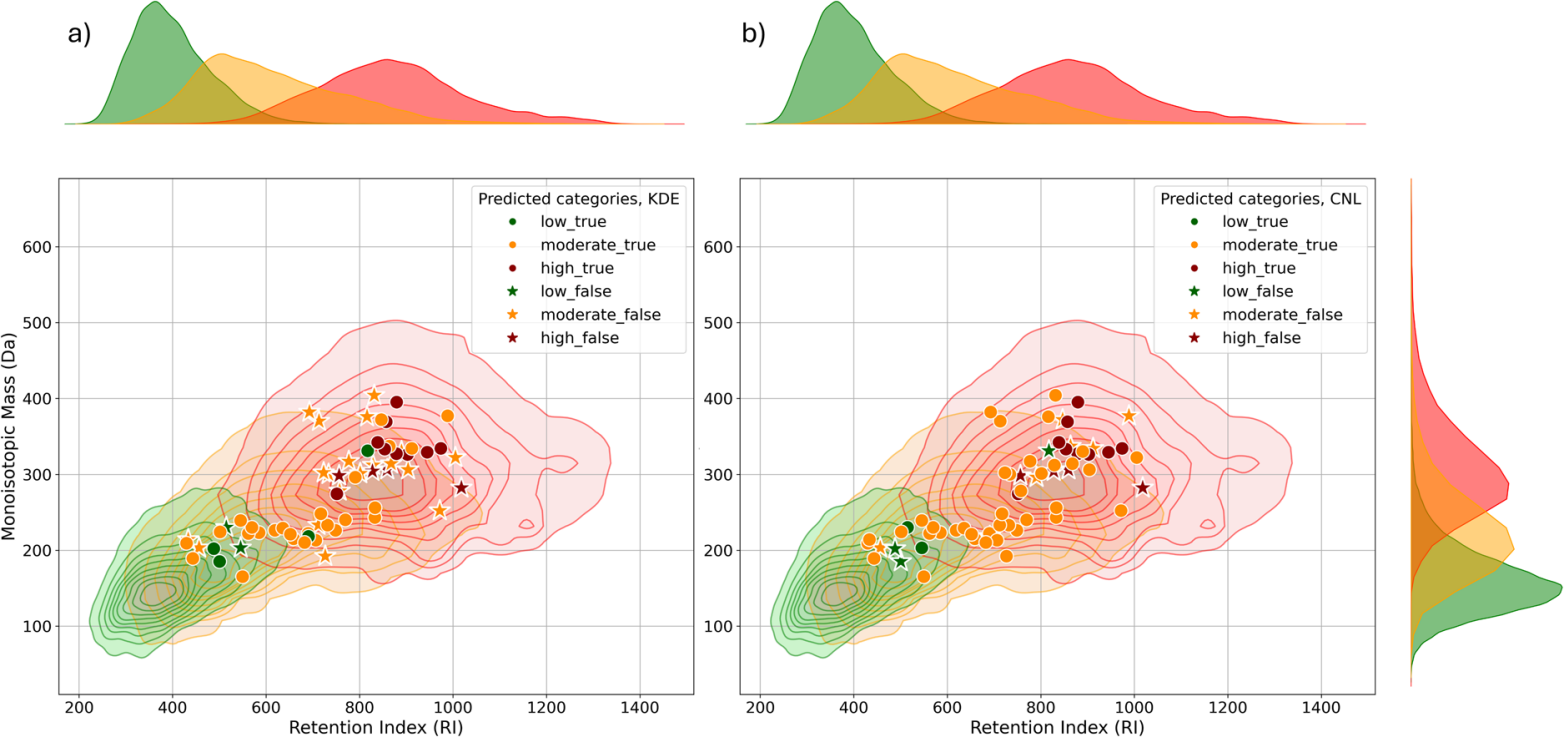
KDE joint plot showing predicted toxicity categories for detected pesticides using (a) a KDE-based model and (b) a CNL-based model. “True” (circle marker) indicates correct predictions, while “False” (star marker) represents incorrect predictions. ″Low,” ″Moderate,” and “High” refer to the predicted toxicity categories.

When applied to the detected pesticides, the CNL-based model achieved an average accuracy of 0.76–0.80, while the KDE model had a lower accuracy of 0.61, Figure 3. Additionally, the accuracy of the CNL model was not significantly influenced by different matrix levels as variations in accuracy across repeated measurements were within 3%, similar to the variation between different matrices. This suggests that the CNL model maintains stability and reliability across diverse experimental conditions, making it particularly useful for complex environmental matrices. We examined chemicals that were misclassified by either the KDE-based or CNL-based models, meaning that they were assigned to a different toxicity category compared to the benchmark (FP-based) model, Figure 3. For instance, Metsulfuron-methyl (INCHIKEY: RSMUVYRMZCOLBH-UHFFFAOYSA-N) was predicted to belong to the high toxicity category by the KDE-based model, while both the CNL-based and FP-based models classified it as moderate toxicity. While there are no measured LC 50 values available for fathead minnows, experimental LC 50 values for Bluegill Sunfish and Rainbow Trout reported in the Pesticide Ecotoxicity Database from the EPA indicate LC 50 values greater than 2 [LOG(mg/L)] for a 96-h exposure. Based on classification criteria, chemicals with LC 50 values above 2 [LOG(mg/L)] and a monoisotopic mass of 381.07 Da typically fall into the moderate or low toxicity categories for fathead minnows. The CNL and FP models, therefore, offered a more accurate classification in this case, reflecting the expected behavior of methylmetsulfuron in aquatic species. On the other hand, thiabendazole (INCHIKEY: WJCNZQLZVWNLKY-UHFFFAOYSA-N) was assigned to the moderate toxicity category by the CNL-based model, while the KDE- and FP-based models classified it as low toxicity. Again, no data were available for fathead minnows, but the LC 50 values for Bluegill Sunfish and Rainbow Trout, as recorded by the EPA, range between −0.25 and 1.8 [LOG(mg/L)]. With an accurate mass of 201.04 Da, this chemical is typically assigned to either moderate or low toxicity categories. The discrepancy in classification highlights the challenges of classifying compounds that fall near the boundary between the toxicity classes. In this case, the CNL model’s assignment of moderate toxicity may be more reflective of the compound’s potential toxicity, given the reported LC 50 values and structural characteristics.

Both data sets used for comparison of the models are relatively small, with 98 and 66 chemicals in the global test set and pesticide mixture data set, respectively. However, they differ in their chemical variability, making their performance assessments complementary. Since the global data set has high chemical and toxicological diversity, the performance of the KDE and CNL models differ only slightly, which is not statistically significant. In contrast, the pesticide mixture data set has lower toxicological variability, with 72% of its chemicals falling into the moderate toxicity category. This results in the highest uncertainty for the KDE-based model as it assigns toxicity solely based on the PDFs of toxicity categories, Figure 3. In comparison, the CNL model incorporates structural information, leading to a more detailed toxicity assessment. Both models performed well on experimental and global test data sets, showing comparable results to the FP-based model for low and high toxicity categories. For moderate toxicity, the CNL-based model yielded better results as it incorporates additional structural information when compared to the KDE model. Due to the fact that the distribution of the moderate toxicity category overlaps with the adjacent categories/distributions, our models face additional difficulties for the chemicals at the edge of the moderate category. A larger data set with a larger portion of chemicals accurately measured at the boundaries of the moderate category may provide the means for higher accuracy models. Overall, the CNL-based model outperformed the KDE-based model in the pesticide data set, demonstrating superior predictive power and more consistent classification.

Several recent studies have focused on linking high-resolution accurate mass tandem mass spectrometry (MS2) data to EC 50 and LC 50 values. These studies primarily aim to predict LC 50 or EC 50 values, which are then converted into toxicity categories.^33−36^ Typically, this process involves converting the spectra into estimated molecular formulas and predicted probabilistic molecular fingerprints using tools like SIRIUS. The generated probabilistic fingerprints are then used for LC 50 or EC 50 prediction. As a result, the performance of these models depends on the accuracy of the molecular formulas and fingerprint predictions. In contrast, our approach bypasses the need for molecular structure prediction by directly linking mass spectral signals to toxicity categories. Instead of using a regression model to predict continuous EC 50 or LC 50 values, we applied a classification algorithm that assigns compounds directly into toxicity categories. By relying solely on chromatographic and fragmentation data, our CNL- and KDE-based algorithms present a unique approach that differs from the structure-dependent models used in previous studies, making direct comparisons challenging.

## Implications

In this study, we showed a novel feature prioritization approach based on predicting toxicity categories by employing two algorithms: a probabilistic KDE-based model and a machine learning-based CNL model. Due to the complex nature of environmental samples and the highly convoluted data, fragmentation information is not consistently available for all features. To mitigate the influence of MS2 signal availability, we developed a KDE-based algorithm, which relies solely on MS1 and retention of the features. On the other hand, the combination of retention, MS1, and fragmentation patterns or CNLs enhances prediction accuracy. Our approach connects chromatographic and fragmentation data directly to toxicity categories, bypassing the need for compound identification, a common requirement in currently available prioritization strategies. Moreover, our models demonstrated accuracy comparable to those that depend on structural information (FP-based models), enabling effective toxicity predictions even for compounds with unknown structures. This makes our models not only valuable for the potential prioritization of unknown features in current NTA studies but also suitable for enhancing retrospective analyses of historical data sets.

One of the current limitations of the developed approach is the limited number of measured toxicity values, retention indices, and high-resolution mass spectra, which reduces the model’s applicability domain. Currently, the model relies solely on RPLC-ESI(+)-HRMS due to the scarcity of available data. Additionally, it is based on data from a single trophic level and acute toxicity. More specific mode-of-action models can give more insights into hazard scores and risk assessment. Another challenge arises from the fact that the model was trained on clean spectra obtained from publicly available spectral databases. In real LC-HRMS measurements, coeluted compounds, instrumental artifacts, and background noise can introduce false-positive fragments, potentially reducing prediction accuracy. As a result, robust and reliable data preprocessing is critical, especially for DIA experiments, to ensure the accuracy of the model.

## Data Availability

The script to perform the calculations is available at 10.5281/zenodo.14188167. The data sets used for the development of the algorithms are available at https://bitbucket.org/viktoriiaturkina/toxmap_prioritization.jl/src/main

## Supporting Information Available

Additional details on acquisition method for pesticide mixture data set (Section S1); distribution on assigned toxicity categories and training, test, and global test set of fish toxicity data set (Figure S1); plotted CompTox data set with predicted toxicity categories (Figure S2); equation for inverse class frequency calculation (Equation S1); regression equation to convert *tr* into *RI* (Equation S2); additional details on model performance (Figure S4–S6, Table S2); and number of fragments matched with suspect screening (Table S3, Figure S7) (PDF)
