# Supplementary material for: Prioritization of Unknown LC-HRMS Features Based on Predicted Toxicity Categories
Source: Environ Sci Technol. 2025 Apr 21;59(16):8004–15. doi: 10.1021/acs.est.4c13026 (PMC12044687; doi:10.1021/acs.est.4c13026)
Supplement: Supplementary file 1 — es4c13026_si_001.pdf [file es4c13026_si_001.pdf]

Supporting information for: Prioritization of unknown LC-HRMS features based on predicted toxicity categories

Viktoriia Turkina,[*,†] Jelle T. Gringhuis,[†] Sanne Boot,[†] Annemieke Petrignani,[†] Garry Corthals,[†] Antonia Praetorius,[‡] Jake W. O'Brien,[¶,†] and Saer Samanipour[*,†,§,‖]

†*Van 't Hoff Institute for Molecular Sciences (HIMS), University of Amsterdam, 1090 GD, Amsterdam, the Netherlands*

‡*Institute for Biodiversity and Ecosystem Dynamics (IBED), University of Amsterdam, 1090 GE, Amsterdam, the Netherlands*

¶*Queensland Alliance for Environmental Health Sciences (QAEHS), The University of Queensland, 20 Cornwall Street, Woolloongabba, QLD, 4102, Australia*

§*UvA Data Science Center, University of Amsterdam, Amsterdam*

‖*Queensland Alliance for Environmental Health Sciences (QAEHS), 20 Cornwall Street, Woolloongabba, QLD, 4102, Australia*

E-mail: v.turkina@uva.nl; s.samanipour@uva.nl

Contents

S1 Pesticide mixture acquisition method **S4**

S1.1 Chemicals S4

S1.2 Sample preparation S4

S1.3 LC-ESI-QTOF S5

S2 Fish toxicity dataset **S6**

S3 CompTox dataset **S7**

S4 Applied equations **S8**

S4.1 Inverse class frequency S8

S4.2 Regression equation S8

S5 Results and discussion **S9**

List of Figures

S1 Fish toxicity datasets represented on MW [Da] and LC50 [LOG(mg/L)] with assigned toxicity categories bases on K-means clustering (a). The plot (b) illustrate the spread across these two dimensions for the training set, test set, and global test set. S6

S2 Scatterplot of CompTox dataset on monoisotopic mass and retention indices dimensions (a) and assigned toxicity categories (b) S7

S3 Regression line between *tr, min* 18 detected in pesticide mixture dataset standards and their predicted *RI*. S8

S4 Confusion matrix for FPs-based model toxicity categories prediction for fish toxicity (a) training and (b) test set and (c) leverage versus difference between measured and predicted toxicity categories. S9
S5 (a)Confusion matrix for KDE-based model toxicity categories prediction for fish toxicity dataset and (b) leverage versus difference between measured and predicted toxicity categories. S10
S6 Confusion matrix for predicting toxicity categories using: (a) an FP-based model, (b) a CNLs-based model, and (c) a KDE-based model, applied to the global test set. S11
S7 Distributions of number of detected fragments in all six measurements of pesticide mixture in three different matrices S12

List of Tables

S1 List of chemicals with manufactures used for pesticide mixture in tea matrix LC-HRMS data acquisition S4
S2 The performance assessment of the CNLs-based classification model. S11
S3 Results of suspects screening reported as a number of detected pesticides (n pesticides), median, 25% quantile (Q25), and 75% quantile (Q75) of number of detected fragments for all pesticides in each sample. S12

S1 Pesticide mixture acquisition method

S1.1 Chemicals

Table S1: List of chemicals with manufactures used for pesticide mixture in tea matrix LC-HRMS data acquisition

Chemical	Manufacturer
Deionized water	Milli-Q Integral 3 unit from MilliporeSigma (Germany)
Acetonitrile (gradient grade forliquid chromatography, LiChrosolv)	MilliporeSigma (Germany)
Ethanol (absolute for analysis, EMSURE)	MilliporeSigma (Germany)
Formic acid ($\geq$ 99%, HiPerSolv CHROMANORM for LC-MS)	VWR Chemicals (Germany)
LC/MS Pesticide Comprehensive Test Mix Kit (PN 5190-0551), 100 mg/L	Agilent Technologies

S1.2 Sample preparation

Black tea Klassik and rooibos tea Rooibos Vanille from Teekanne, Germany were used to prepare a tea extract. One tea bag of each type with a dry weight of 1.75 g each were sonicated for 25 min in 100 ml of a water/ethanol solution (50%:50%, v/v). The obtained extract was filtered through a Captiva syringe filter (0.2 μm pore size, regenerated cellulose, Agilent Technologies).

Three blank solutions serving as diluent and negative controls were prepared as either filtered water/ethanol solution (50%:50%, v/v, Blank A) or filtered tea extract that was further diluted 1:10 (Blank B) or 1:100 (Blank C) with Blank A. The eight submixtures of the comprehensive pesticide mix kit were kept initially separate and diluted with Blank

A to final analyte concentrations of 100 μg/L. All eight pesticide submixtures were pooled together and diluted with Blank A, B, or C to final concentrations of 100 μg/L.

S1.3 LC-ESI-QTOF

The samples were analysed with a 1290 Infinity II LC system (Agilent Technologies, Germany), consisting of a binary pump (G7120A), an autosampler (G7129B), and a column oven (G7116B). The autosampler's temperatue was set at room temperature (ca. 20 °C) and the injection volume was 1 μL. The separation was performed on a Poroshell EC-C18 column (2.1 mm × 150 mm, 2.7 μm, Agilent Technologies) at a constant flow rate of 0.5 mL/min and a column temperature of 40 °C. The mobile phases were water + 0.1% formic acid (A) and acetonitrile + 0.1% formic acid (B). The following gradient program was used for the separation of analytes: at 0 min, 95% A; at 1 min, 95% A; at 21 min, 5% A; at 23 min, 5% A; at 23.1 min, 95% A; and at 28 min, 95% A.

The LC system was connected to a quadrupole time-of-flight (Q-TOF) mass spectrometer (G6546A, Agilent Technologies), equipped with an electrospray ionization (ESI) source using the Dual Spray Agilent Jet Stream technology. The Q-TOF was operated in the high-resolution mode for the low (m/z 1700) mass range. The acquisition rate was set to 6 Hz performing all ion full scan measurements at alternating collision energies of 0, 20, and 40 eV. Data were always recorded for a mass range of m/z 50–1200 in profile storage mode. Ionization was performed in the positive mode and the ESI source was operated under the following conditions: a drying gas temperature of 225 °C, a drying gas flow of 12 L/min, a sheath gas temperature of 350 °C, a sheath gas flow of 11 L/min, and a nebulizer pressure of 35 psi. The capillary and nozzle voltages were kept at 3500 and 500 V, respectively. A reference solution, containing purine and hexakis(1H,1H,3H-tetrafluoropropoxy)phosphazine (HP-0921), was continuously supplied to the second sprayer of the ESI source using an isocratic pump (G7110B, Agilent Technologies, Germany).

S2 Fish toxicity dataset

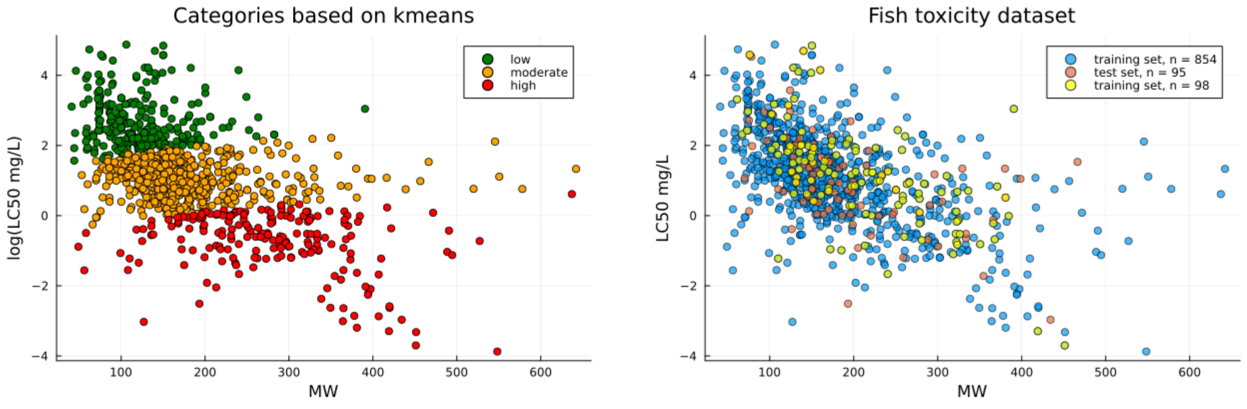

(a) (b)

Figure S1: Fish toxicity datasets represented on MW [Da] and LC50 [LOG(mg/L)] with assigned toxicity categories bases on K-means clustering (a). The plot (b) illustrate the spread across these two dimensions for the training set, test set, and global test set.

S3 CompTox dataset

(a)

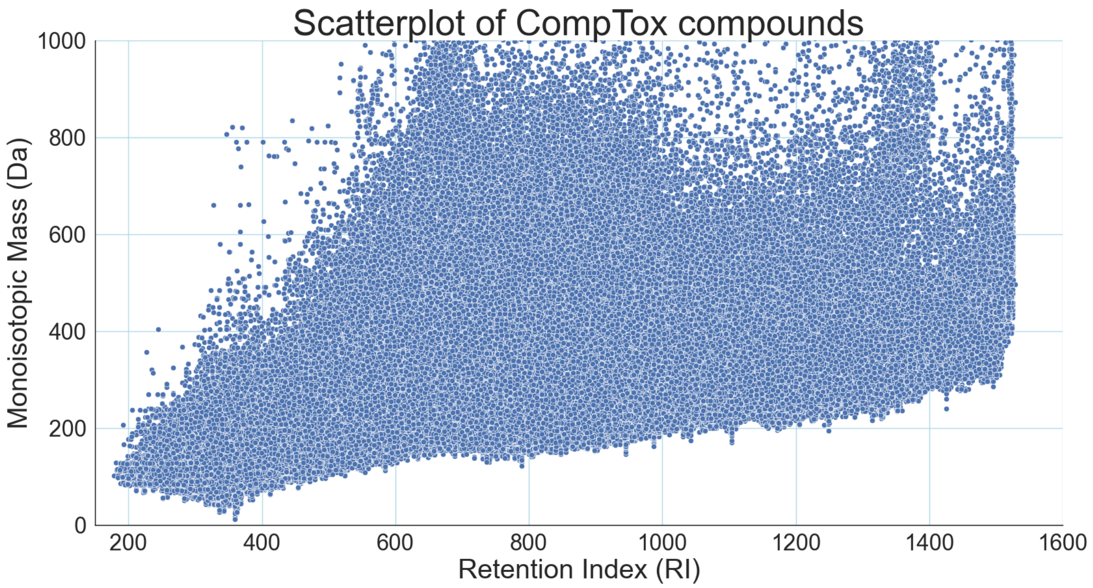

(b)

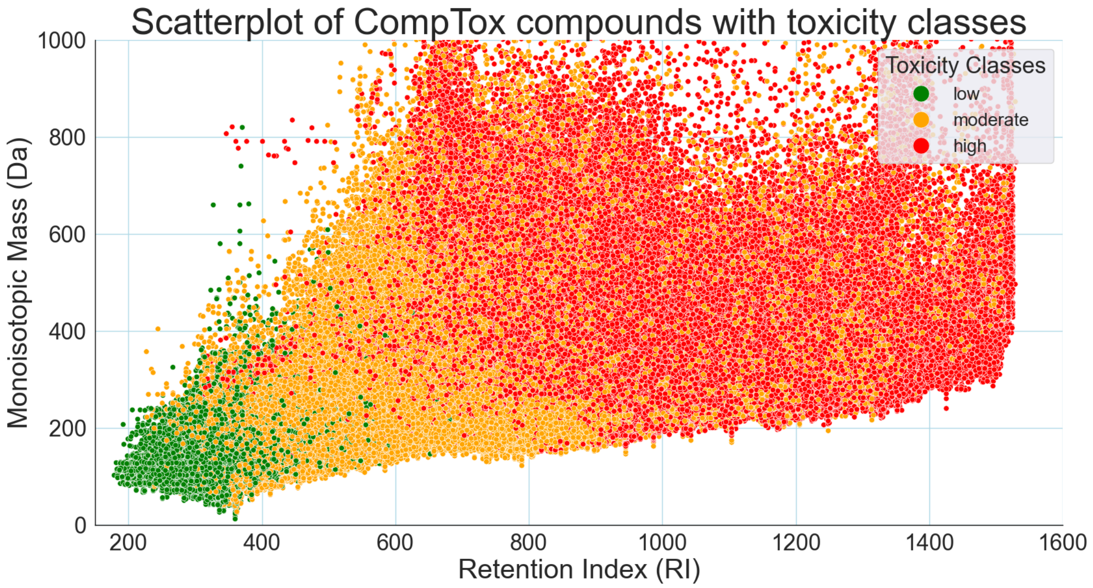

Figure S2: Scatterplot of CompTox dataset on monoisotopic mass and retention indices dimensions (a) and assigned toxicity categories (b)

S4 Applied equations

S4.1 Inverse class frequency

The equation to compute the inverse class frequency to assign higher weights to underrepresented classes in the training set.

$$v = \frac{n_{\text{samples}}}{n_{\text{cats}} \cdot count_{\text{cats}}} \tag{S1}$$

Where: v - inverse toxicity category frequency; n_{samples} - number of compounds in the training set; n_{cats} - number of toxicity categories; $count_{\text{cats}}$ - the number of occurrences of each toxicity category in the training set;

More detailed information can be found at `https://scikit-learn.org/stable/modules/generated/sklearn.ensemble.RandomForestClassifier.html`.

S4.2 Regression equation

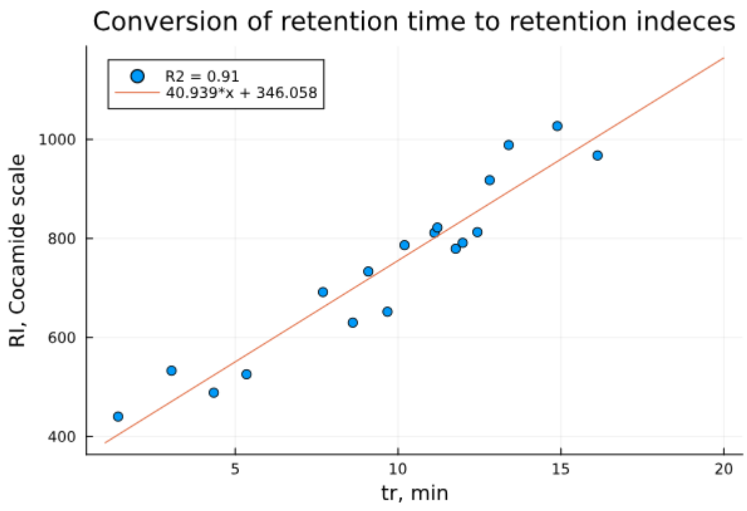

Figure S3: Regression line between *tr, min* 18 detected in pesticide mixture dataset standards and their predicted *RI*.

$$RI = 40.939 \cdot tr + 346.058 \tag{S2}$$

Regression equation to convert *tr, min* of detected standards in the pesticide mixture dataset into their *RI*.

S5 Results and discussion

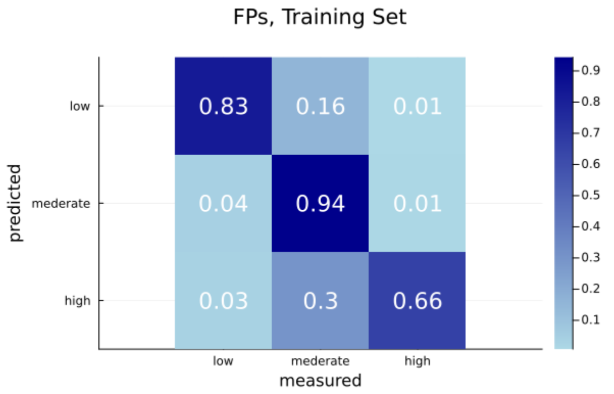

(a)

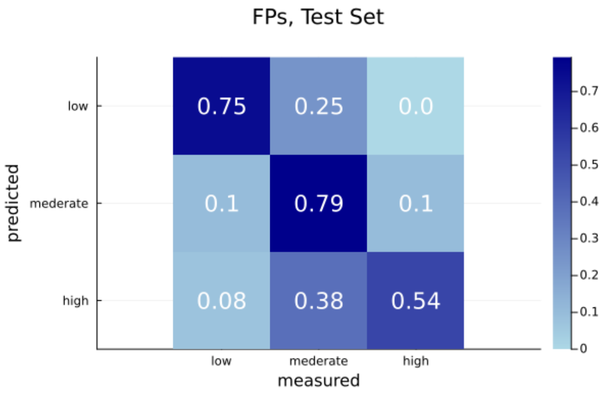

(b)

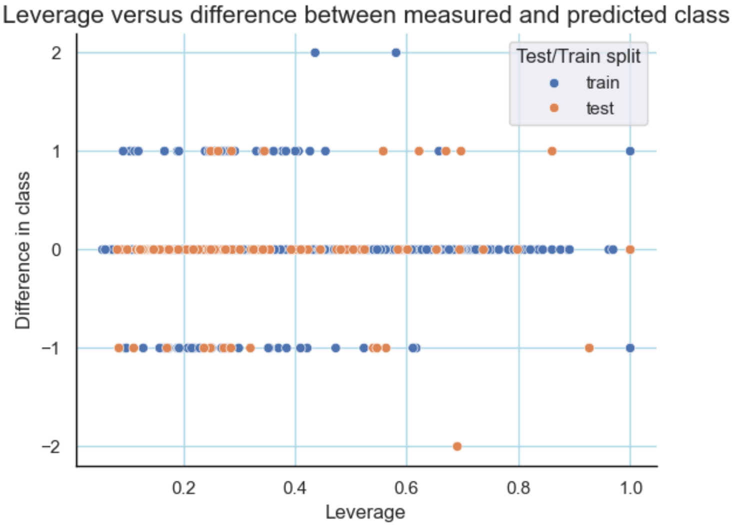

(c)

Figure S4: Confusion matrix for FPs-based model toxicity categories prediction for fish toxicity (a) training and (b) test set and (c) leverage versus difference between measured and predicted toxicity categories.

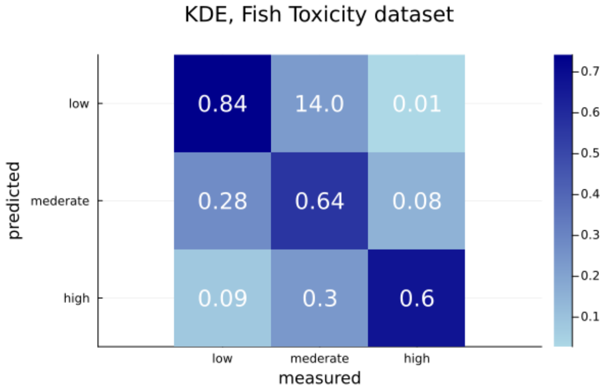

(a)

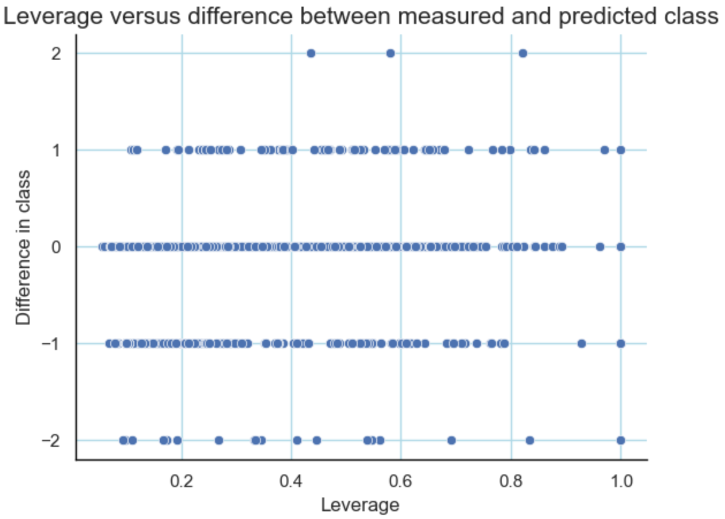

(b)

Figure S5: (a)Confusion matrix for KDE-based model toxicity categories prediction for fish toxicity dataset and (b) leverage versus difference between measured and predicted toxicity categories.

Table S2: The performance assessment of the CNLs-based classification model.

	Class	Precision	Recall	F1-Score	Support	Accuracy
Training set	Low	0.52	0.95	0.67	2821	0.76
	Moderate	0.96	0.63	0.76	11404	
	High	0.72	0.96	0.82	4610	
Test set	Low	0.50	0.91	0.65	331	0.72
	Moderate	0.90	0.60	0.72	1258	
	High	0.68	0.90	0.77	504	

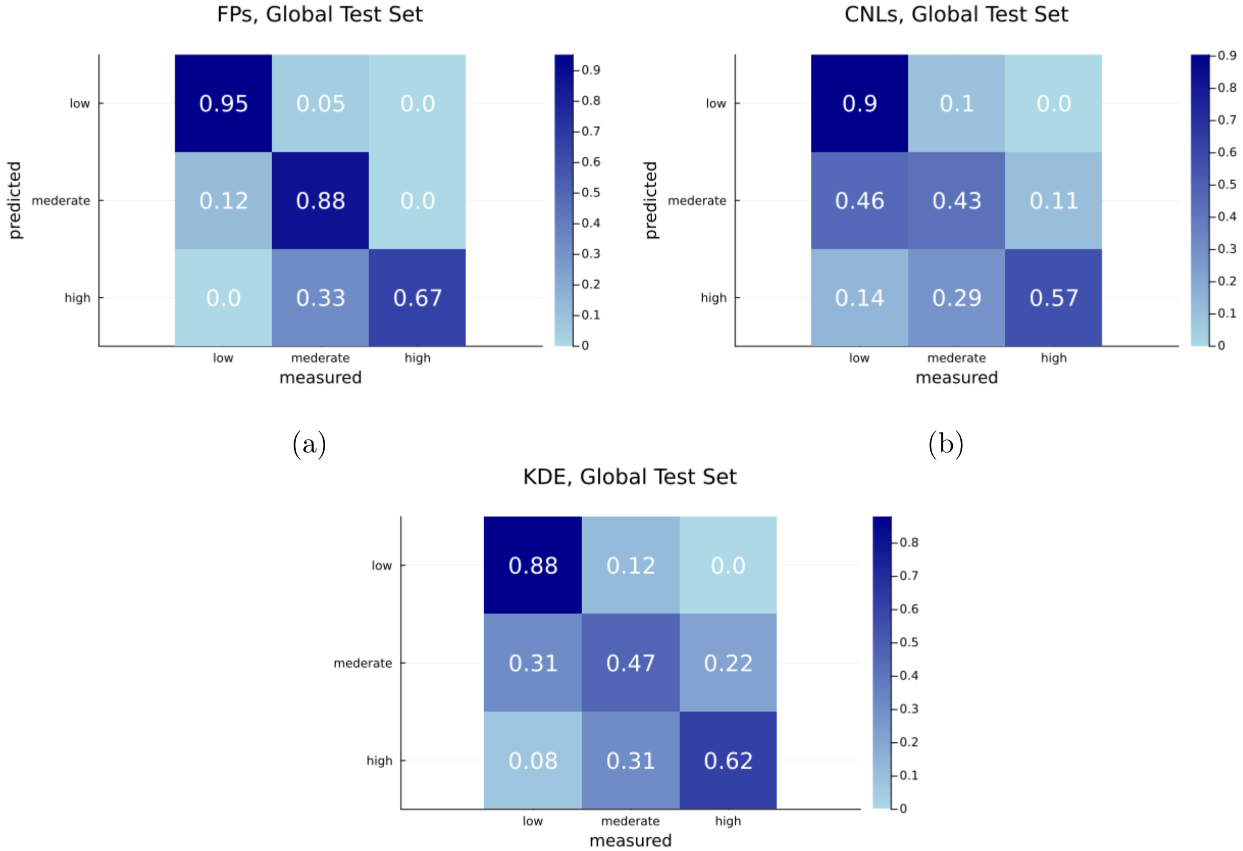

(a) (b) (c)

Figure S6: Confusion matrix for predicting toxicity categories using: (a) an FP-based model, (b) a CNLs-based model, and (c) a KDE-based model, applied to the global test set.

Table S3: Results of suspects screening reported as a number of detected pesticides (n pesticides), median, 25% quantile (Q25), and 75% quantile (Q75) of number of detected fragments for all pesticides in each sample.

Matrix	n pesticides	median	Q25	Q75
No tea rep1	152	13	7	23
No tea rep2	145	12	7	21
$\times$100 dilution rep1	154	13	7	22
$\times$100 dilution rep2	141	13	7	23
$\times$10 dilution rep1	148	12	7	22
$\times$10 dilution rep2	155	12	6	21.25

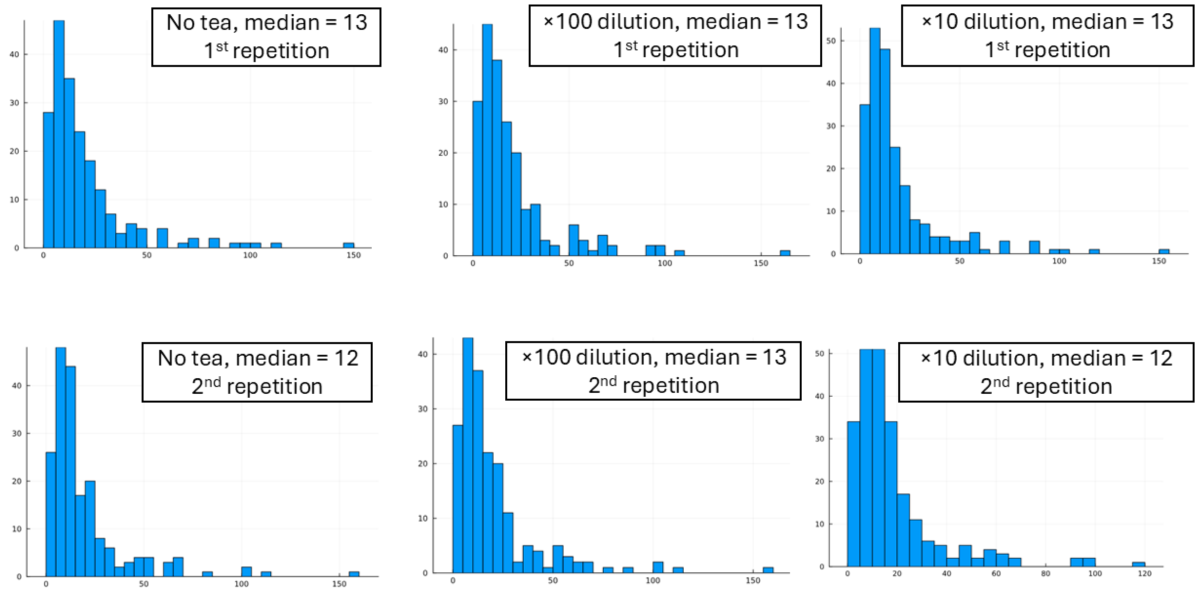

Figure S7: Distributions of number of detected fragments in all six measurements of pesticide mixture in three different matrices
